# Iodine nutrition status and thyroid autoimmunity during pregnancy: a cross-sectional study of 4635 pregnant women

**DOI:** 10.1186/s12937-022-00760-6

**Published:** 2022-01-29

**Authors:** Xiao Chen, Chunfeng Wu, Zhengyuan Wang, Chunxiang Wu, Yan Guo, Xiaoxia Zhu, Yan Ping Hu, Zehuan Shi, Qi Song, Xueying Cui, Jin Su, Jiajie Zang

**Affiliations:** 1 Shanghai Putuo District Center for Disease Control and Prevention, Shanghai, 200033 China; 2 Shanghai Municipal Center for Disease Control and Prevention, Shanghai, 200336 China

**Keywords:** Mild iodine deficiency, thyroid autoimmunity, pregnancy, urinary iodine concentration

## Abstract

**Background:**

Pregnant women in Shanghai have long been at risk for mild iodine deficiency. Because thyroid autoimmunity in pregnant women can lead to premature birth and miscarriage as well as neurodevelopmental deficits in the fetus, the aim of this study was to explore the association of iodine nutrition status with thyroid antibodies during pregnancy.

**Methods:**

A pregnancy-birth cohort was conducted including 4635 pregnant women in Shanghai, China. The eligible participants underwent a face-to-face interview and completed questionnaire surveys to collect baseline information and diet intake. Spot urine samples were collected to test urine iodine. Thyroid antibodies including thyroid peroxidase antibodies (TPOAb), thyroglobulin antibodies (TgAb) and thyrotrophic antibodies (TRAb) were tested. Single-factor analysis and logistic regression were used to evaluate the association between iodine status and thyroid autoimmunity during pregnancy.

**Results:**

The median urinary iodine excretion level in the sample was 138.14 μg/L (interquartile range [IQR] 80.90–219.00 μg/L). Among all the subjects, 25.9% consumed non-iodized salt, 54.5% had iodine deficiency, and 31.0% had thyroid autoimmunity. The proportion of patients with iodine deficiency was significantly higher among those who consumed non-iodized salt (36.9% vs. 33.1%; *p* = 0.04). After adjusting for age, educational status, former smoker status, former drinker status, first pregnancy, and previous thyroid disease, non-iodized salt (odds ratio [OR] = 1.394 [confidence interval, CI, 1.165–1.562]; *p* = 0.003), iodine-rich food (OR = 0.681 [CI 0.585–0.793]; *p* = 0.003), iodized nutritional supplements (OR = 0.427 [CI 0.347–0.526]; *p* = 0.003), were found to be individually associated with thyroid autoimmunity in all participants. The results of the multivariable restricted cubic spline regression analysis showed a non-linear relationship between the continuous change in iodine intake and thyroid autoimmunity (*p* = 0.019). Participants with iodine deficiency (urinary iodine concentration, UIC,< 100 μg/L) had an increased risk of testing positive for thyroid antibodies (TPOAb/TgAb/TRAb[+]; OR = 1.324 [CI 1.125–1.559]; *p* < 0.001). Moreover, this associated existed even after removing participants with previous thyroid disease.

**Conclusion:**

Inadequate iodine nutrition in pregnant women is an independent risk factor for thyroid autoimmunity in Shanghai. It’s important to maintain the adequate iodine status in pregnant women.

## Introduction

Thyroid autoimmunity is one of the most prevalent organ-specific autoimmune diseases. It is usually characterized by the presence of antibodies against thyroid-specific components such as thyroglobulin, thyroid peroxidase, and thyrotrophic receptor antigen. The prevalence of autoimmune thyroid disease has been estimated to be 5% in the general population, and the percentage of patients with thyroid antibodies without clinical manifestation may be even higher [1, 2]^.^ A recent study by Shan et al. showed a significantly increased prevalence of thyroid peroxidase antibodies (TPOAb) and thyroglobulin antibodies (TgAb) positivity compared to the corresponding data for 2015 and 1999 (TPOAb: 11.5% vs. 9.81%; TgAb: 12.6% vs. 9.09%) [3]. Further, thyroid autoimmunity was much more common among women than in men, with the female: male ratio ranging from 5:1 to 10:1. This sex difference may be related to changes in antibodies during pregnancy [3–5]. Thyroid autoimmunity has been reported to be the main cause of hypothyroidism during pregnancy [6].

Thyroid hormones are required for growth, brain development, metabolism, and energy balance. Pregnancy results in an increased demand for thyroid hormones. The development of maternal thyroid disorders during early pregnancy can influence the pregnancy outcome and fetal development. Recently, many studies have shown that positivity for thyroid autoantibodies in pregnant women can lead to premature birth and miscarriage as well as neurodevelopmental deficits in the fetus [7, 8]. Therefore, thyroid autoimmune diseases during pregnancy are receiving widespread attention.

Epidemiological data show that genetic susceptibility, environmental factors, including nutritional factors (such as diet, iodine nutrition, and medications), immune disorders (e.g., infection), smoking, and alcohol consumption contribute to the development of thyroid autoimmunity. Among environmental factors, iodine appears the most important [4]. Iodine is required for thyroid hormone synthesis, and adequate production of thyroid hormones is essential for brain and body development. The causal relationship between iodine and thyroid autoimmunity remains unclear. Excessive iodine intake and iodine deficiency might enhance or initiate the autoimmune process in the thyroid gland [9].

China used to be an iodine-deficient country with a high prevalence of iodine deficiency disorders (IDDs). Since 1996, China has implemented the strategy of universal salt iodization (USI) to eliminate iodine deficiency. After two decades of mandatory universal salt idolization, the goal of eliminating iodine deficiency (ID) has been successfully achieved in China [10]. However, the prevalence and spectrum of thyroid disorders have increased, indicating possible adverse effects of increased iodine intake [3, 10]. These findings are consistent with those of studies conducted in the United States and Europe [11, 12]. Shanghai is iodine deficiency in the natural environment. Iodized salt is the main vehicle for iodine carrier Shanghai, and the iodine status in vulnerable populations in the region has been monitored every years. Recent monitoring results showed that pregnant women had a moderate level of iodine deficiency in recent 10 years [13, 14]. Studies conducted in other countries and regions of the world, such as the United Kingdom [15], Australia [16], Spain [11], Netherlands [12] and the United States [11], also found that local pregnant women are iodine deficient. The issue of maternal iodine nutrition has attracted worldwide attention.

Recent studies in Shanghai has shown that the current dietary iodine intake is insufficient in pregnant women [13]. Meanwhile, that study has not reported the prevalence of the thyroid antibodies positivity in pregnant women in Shanghai. Therefore, the general objective of this study was to investigate the prevalence of thyroid autoantibodies and their associations with iodine intake and iodine status in 4635 pregnant women in Shanghai, China, a coastal area.

## Materials and Methods

### Study population

**Fig. 1:**
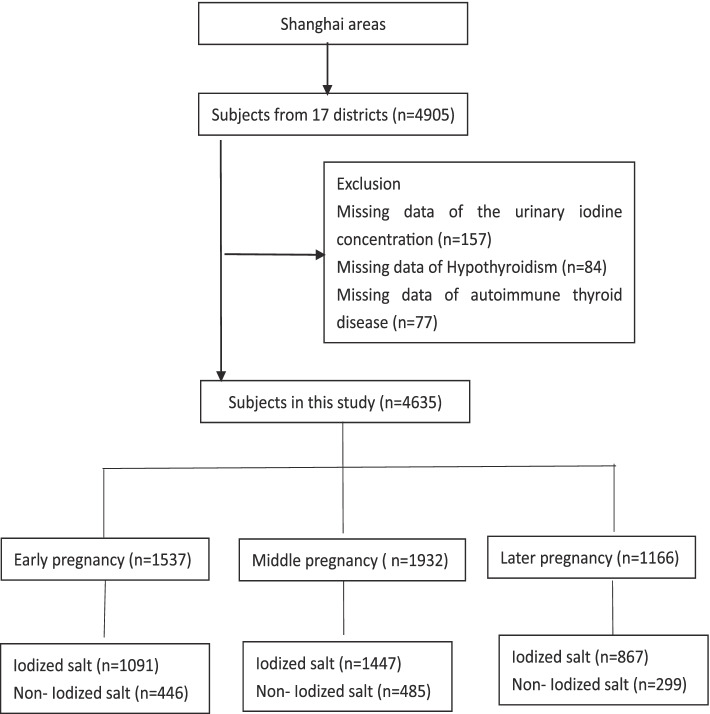
Flow chart of the inclusion and exclusion of participants

This study was based on the iodine status in pregnancy and offspring health cohort and was conducted from April to October 2017. A multistage, stratified random sampling method was used to obtain a representative sample. In light of the sample size and the number of pregnant women in each administrative district in 2016 each district was divided into five sections, a street was randomly selected from each section, and an equal number of pregnant women were selected from each section. From April to October 2017, a total of 4905 pregnant women were recruited. Exclusion criteria were subjects with chronic disease conditions to eliminate potential influence, such as pre-diagnosed diseases in kidney, cardiovascular system, endocrine and other systems that might affect the iodine metabolism and excretion. Participants whose laboratory results were missing, including those for urinary iodine concentration (*n* = 157), hypothyroidism (*n* = 84), and thyroid autoimmunity (*n* = 77) were excluded. According to the gestational week of pregnancy, the participants were divided into early pregnancy (≤12 weeks), middle pregnancy (13 ~ 28 weeks), and later pregnancy (≥29 weeks) groups [17]. Ultimately, the early pregnancy, middle pregnancy, and later pregnancy groups included 1537, 1932, and 1166 participants, respectively (Fig. 1). The study was approved by the Ethics Committee of Shanghai Center for Disease Control and Prevention (SCDC) and was performed in accordance with the Declaration of Helsinki. Before data collection, written informed consent was obtained from all participants.

### Measurements

All participating pregnant women received questionnaire surveys and provided 10 ml of whole blood samples and 20 ml of random urine samples during pregnancy at the same time.

A structured questionnaire was designed to obtain general information, which included questions on demographic characteristics, lifestyle-related risk factors, fertility history, a personal or family history of thyroid disease, and food frequency questionnaire (FFQ). FFQ included iodine-containing food and iodine-containing nutrient supplements and measuring the frequency and amount of foods consumed including nutrient supplement by participants over the past 3 months [18]. The questionnaire was administered face-to-face by interviewers from local community health centers who received a standard training course on the recording of dietary information. The interviewers read aloud the standard portion size of each food item for every question. Visual aids relating to the standard portion sizes were shown to the participants [19].

### Collection of general information

Participants aged higher than 35 years were considered an advanced maternal age. Education status was divided into three categories: 9-year compulsory education, senior high school and college, and bachelor’s degree and higher. The family income categories were as follows: less than 100,000, 100,000 to 200,000, and more than 200,000 Yuan [20]. Participants who smoked and drank alcohol before and after pregnancy, excluding who occasionally attempted, were defined as “Former smoker” and “Former drinker” respectively. “First pregnancy” meant that the pregnancy at the time of the study was the participant’s first pregnancy, excluding previous miscarriages and stillbirths. “Iodine-containing food” included nori, seaweed, wakame, shrimp, and mussel. “Previous thyroid disease” was defined that participants had suffered from thyroid diseases including thyromegaly, thyroid nodule, hyperthyroidism, hypothyroidism, Craves disease, Hashimoto’s thyroiditis, which diagnosed by qualified doctors in second-level or higher hospitals.

### Types of salt used by the participants

The question “Currently, which type of salt is consumed by your family?” was used to collect information about the type of salt consumed. The participants were asked to select from three options as a response for this question: (i) iodized salt, (ii) non-iodized salt, and (iii) both. If a participant chose “both,” the participant was considered to consume “iodized salt.”

### Total iodine intake calculation

The total iodine intake was estimated from all main food sources including iodine-rich food, iodine-containing nutrient supplements. The iodine content of each food item was calculated using the 2013 China Food Composition Database [21]. The iodine content of iodine-containing nutrient supplement items was calculated using the product manual. The frequency of food intake was measured in terms of four categories: “times per day”, “times per week”, “times per month”, “times per 3 month”. The average food recordings were conversed according to the requirements of each food (raw weight, edible weight, dry weight, fresh weight, etc.) and the conversion rate. Similar foods were counted together. The iodine intake from iodine-rich food and supplements was determined as follows: iodine concentration in food (μg/g) × serving size (g) × frequency factor. If a pregnant woman consumed household salt during the investigation, the quantity of iodine obtained from the salt was determined by the titration method, and the salt intake were weighed before and after the 7 survey days. The iodine intake from salt was calculated as: iodine concentration in salt (mg/kg) × salt consumption(g/day).

### Urine sample collection, testing, and evaluation

Participants collected 20 ml of random urine to test urine iodine during survey. All samples were immediately stored in a 4 °C refrigerator, transported to the designated laboratory, which was a qualified testing laboratory and inspecting organization that complied with international standards ISO/IEC17025 and ISO/IEC 17020, within 6 h, and then stored at − 80 °C until analysis. The acid digestion method (As3 + −Ce4 + catalytic spectrophotometry [22]) was applied to test each iodine urinary sample in the Shanghai Municipal Center for Disease Prevention and Control. Internal quality control samples for urinary iodine concentration (UIC) were obtained from the Chinese National Iodine Deficiency Disorders Reference Laboratory.

Iodine status was estimated by the recommended WHO/UNICEF/ICCIDD criteria from 2007 and other published criteria and was categorized as follows: severe insufficiency (UIC less than 50 μg/L); moderate insufficiency (UIC more than 50 μg/L and less than 100 μg/L); mild insufficiency (UIC more than 100 μg/L and less than 150 μg/L); adequate (UIC more than 150 μg/L and less than 249 μg/L); or excessive or more than the requirement (UIC more than 250 μg/L) [23].

### Thyroid antibody testing and evaluation

Antibody levels againstserum thyrotropin (TSH), thyroid peroxidase (TPO), thyroglobulin (Tg), and thyrotrophic receptor (TR) were measured in all participants. The laboratory reference ranges obtainedusing a Cobas Elecsys 602 (Roche Diagnostics, Switzerland) system were used in this study: TSH, 0.27–4.2 mIU/L; TPO antibody (TPOAb), 0–34 IU/mL; TgAb, 0–115 IU/mL; and TRAb, 0–1.75 IU/L. Positive TPOAb and TgAb were defined as TPOAb> 34 IU/mL and TgAb> 115 IU/mL, respectively [24]. Patients who showed positive findings for at least one of the three thyroid antibody indicators were defined as TPOAb/TgAb/TRAb positive (+) and the others were defined as TPOAb/TgAb/TRAb negative (−). Hypothyroidism was defined as elevation in the serum levels of TSH.

### Statistical analysis

Statistical analysis was performed using SAS 9.4 software and R version 4.0.2. General characteristics were summarized as median with inter-quartile range (IQR) for continuous variables or as proportion for categorical variables. Mann-Whitney’s U-test and the Kruskal-Wallis test were used for comparisons of two or more groups of non-normal data. Pearson’s chi-squared tests were performed to compare categorical variables. Restricted cubic spline (RCS) functions were used to detect the possible nonlinear dependency of the relationship between the risk of thyroid autoimmune diseases and iodine intake, the package of rms of R was used to perform RCS.

The adjusted associations of type of salt, iodine-rich food consumption, iodized nutritional supplements with thyroid autoimmunity were analyzed with binary logistic regression analyses. The results were expressed as odds ratios (OR) and confidence intervals (CIs) for the proportion of thyroid autoimmunity positive to negative. The following variables were selected as covariates: age, educational status, former smoker, former drinker, first pregnancy, and previous thyroid disease. Educational level is an important socioeconomic covariate and is commonly adjusted in epidemiological studies. Alcohol consumption history was adjusted because emerging evidence has suggested that alcohol is a modulator of the immune system, and alcohol consumption may protect against autoimmune hypothyroidism [4]. Previous thyroid disease could affect thyroid antibody abnormalities in this study; therefore, it was included in the multivariable analyses as a covariate.

## Results

### The type of salt used and UIC in the population

**Fig. 2:**
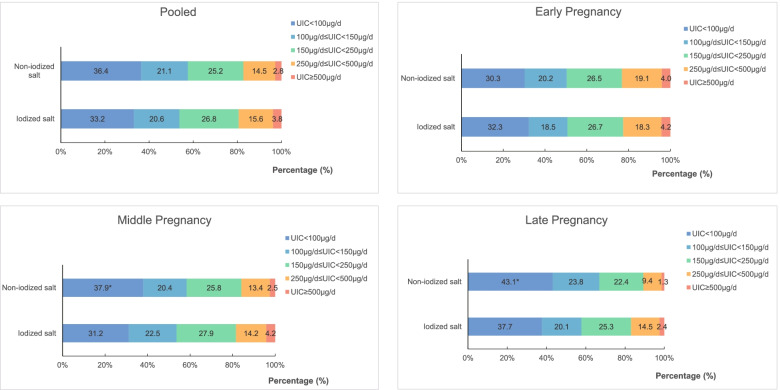
Gestational weeks distribution of urinary iodine concentration stratified by the type of salt used. *p < 0.05 versus iodized salt group

The distribution of UICs in the study population is shown in Fig. 2. The median urinary iodine excretion in the sample was 138.14 μg/L (IQR 80.90–219.00 μg/L). Urinary iodine measurements indicative of moderate ID (UIC ≤ 100 μg/L) and mild ID (100 μg/L < UIC < 150 μg/L) were present in 33.9 and 20.6% of the study population. Meanwhile, 15.4 and 3.7% of the population showed more than adequate (250 μg/L ≤ UIC < 500 μg/L) and excess iodine intake (UIC ≥ 500 μg/L), respectively. In addition, participants who consumed non-iodized salt had a significantly higher proportion of UIC < 100 μg/L (36.9% vs. 33.1%; *p* = 0.04); this was true for participants in middle (37.9% vs. 31.1%; *p* = 0.018; Fig. 2) and late (45.3% vs. 37.1%; *p* = 0.015; Fig. 2) pregnancy as well.

### General characteristics and thyroid disease spectrum stratified by the type of salt used

Table 1 shows general characteristics and the thyroid disease spectrum stratified by the type of salt used. The study population consisted of 4635 pregnant women with a mean ± SD age of 29.2 ± 4.9 years, of which 25.9% consumed non-iodized salt. In comparison with those who consumed iodized salt, subjects who consumed non-iodized salt showed significantly lower UIC (*p* < 0.001); this was true in middle pregnancy (*p* < 0.001) and late pregnancy (*p* < 0.001). Meanwhile, subjects who consumed non-iodized salt had higher educational level (*p* < 0.001), economic income level (*p* < 0.001), first pregnancy rate (*p* < 0.001), previous thyroid disease rate (*p* < 0.001), and elderly maternal rate (*p* = 0.017). Pregnant women taking non-iodized salt in this survey are more likely to take iodine-containing nutritional supplements, and the opposite is true for iodine-rich foods (*p* < 0.001). The prevalences of TPOAb/TgAb/TRAb (+) and TPOAb(+) were significantly higher in the non-iodized salt group compared to the iodized group. Without previous thyroid disease or in different pregnancy groups, those associations still existed.

**Table 1:** General Characteristics and Thyroid Disease Spectrum Stratified by the Type of Salt Used, n(%)

	Pooled		Early pregnancy		Middle pregnancy		Later pregnancy	
	Iodized salt	Non-iodized salt	Iodized salt	Non-iodized salt	Iodized salt	Non-iodized salt	Iodized salt	Non-iodized salt
***N***	3436	1199	1118	419	1439	493	879	287
**Age, years**	29 (26–32)	29 (27–33)	29 (27–32)	30 (27–39)	28 (26–32)	29 (26–33)	28 (26–32)	30 (27–33)
≥ 35	450 (13.10)	191 (15.93)*	160 (14.39)	65 (15.25)	168 (11.67)	75 (15.21)*	122 (13.88)	51 (17.77)*
**Education status** n(%)								
≤ 9 years	1219 (35.50)	239 (20.08)*	318 (28.20)	54 (13.01)*	568 (39.50)	109 (22.29)*	336 (38.23)	76 (26.57)*
Senior high school and college,	856 (24.93)	283 (23.78)*	310 (27.75)	95 (22.89)*	362 (25.17)	123 (25.15)*	184 (20.93)	65 (22.73)*
Bachelor’s degree and higher	1359 (39.57)	668 (56.13)*	492 (44.05)	54 (64.10)*	508 (35.33)	257 (52.56)*	359 (40.84)	145 (50.70)*
**Occupation status**								
Mental	2724 (79.28)	979 (81.65)	873 (78.09)	331 (79.00)	1151 (79.99)	413 (83.77)	700 (79.64)	235 (81.88)
Physical	712 (20.75)	220 (18.35)	245 (21.91)	88 (21.00)	288 (20.01)	80 (16.23)	179 (20.36)	52 (18.12)
**Family income per person last years, yuan**								
< 100,000	667 (19.46)	153 (12.88)*	173 (15.49)	41 (9.93)*	300 (20.92)	71 (14.52)*	194 (22.12)	41 (14.43)*
100,000–200,000	1445 (42.15)	456 (38.38)*	471 (42.17)	158 (38.26)*	608 (42.40)	193 (39.47)*	366 (41.73)	105 (36.71)*
≥ 200,000	1316 (38.39)	579 (48.74)*	473 (42.35)	214 (51.82)*	526 (36.68)	225 (46.01)*	317 (36.15)	140 (48.95)*
**Former smoker**	81 (2.36)	37 (3.11)	27 (2.42)	14 (3.39)	38 (2.65)	15 (3.06)	16 (1.82)	8 (2.79)
**Former drinker**	355 (10.37)	125 (10.56)	110 (9.87)	44 (10.68)	165 (11.50)	52 (10.68)	80 (9.15)	29 (10.18)
**First pregnancy**	1589 (46.25)	662 (55.21)*	536 (47.94)	257 (64.34)*	638 (44.34)	254 (51.52)	415 (47.21)	151 (52.61)
**Previous thyroid disease**	290 (8.44)	217 (18.10)*	104 (9.30)	95 (22.67)*	120 (8.34)	84 (17.04)*	66 (7.51)	38 (13.24)*
**Consumed iodine-rich food in the past 3 months**	2747 (79.95)	923 (76.98)*	858 (76.74)	298 (71.12)*	1175 (81.65)	388 (78.70)	714 (81.23)	237 (82.58)
**Consumed iodized nutritional supplements in the past 3 months**	481 (14.00)	237 (19.77)*	145 (12.97)	64 (15.27)*	200 (13.90)	100 (20.28)*	136 (15.47)	73 (25.44)*
**UIC, μg/L**	141.00 (83.38–221.35)	129.53 (74.01–209.08)*	146.90 (85.80–234.17)	151.15 (89.80–239.17)	141.18 (87.44–218.70)	125.00 (70.56–212.57)*	129.17 (71.78–203.36)	111.51 (58.66–168.35)*
**Hypothyroidism**	91 (2.67)	30 (2.44)	27 (2.47)	13 (2.91)	40 (2.76)	7 (1.44)	24 (2.77)	10 (3.34)
**TPOAb(+)**	439 (12.89)	253 (20.57)*	136 (12.47)	93 (20.85)*	182 (12.58)	108 (22.27)*	121 (13.96)	52 (17.39)
**TgAb(+)**	305 (8.96)	140 (11.38)	123 (11.27)	70 (15.70)	122 (8.43)	49 (10.10)	60 (6.94)	21 (7.02)
**TRAb(+)**	505 (14.90)	201 (16.57)	152 (13.94)	77 (17.58)	223 (15.43)	84 (17.36)	130 (15.22)	40 (13.75)
**TPOAb/TgAb/TRAb(+)**	995 (29.22)	444 (36.10)*	305 (27.96)	160 (35.87)*	430 (29.22)	187 (38.56)*	260 (29.99)	97 (32.44)
**TPOAb(+)(without previous thyroid disease)**	376 (12.00)	173 (17.40)*	107 (10.71)	53 (15.63)*	163 (12.24)	79 (19.95)*	106 (13.20)	41 (15.83)
**TgAb(+) (without previous thyroid disease)**	259 (8.27)	83 (8.35)	102 (10.21)	38 (11.21)	104 (7.81)	30 (7.58)	53 (6.61)	15 (5.79)
**TRAb(+) (without previous thyroid disease)**	467 (14.97)	166 (16.89)	143 (14.31)	60 (17.86)	202 (15.19)	73 (18.48)	122 (15.44)	33 (13.10)
**TPOAb/TgAb/TRAb(+)(without previous thyroid disease)**	890 (28.40)	335 (33.70)*	270 (27.03)	110 (32.45)	386 (28.98)	145 (36.62)*	234 (29.14)	80 (30.89)

Data are summarized as the median values (inter-quartile range) for continuous variables, or as numbers with proportions for categorical variables. Urine iodine concentration, UIC

*p < 0.05 versus the iodized salt group

### General characteristics stratified by TPOAb/TgAb/TRAb positivity

General characteristics in terms of TPOAb/TgAb/TRAb positivity are presented in Table 2. A total of 31.0% of subjects showed TPOAb/TgAb/TRAb(+) status. Compared to subjects in the TPOAb-, TgAb-, and TRAb-negative (TPOAb/TgAb/TRAb [−]) group, those with thyroid autoimmunity had significantly lower UIC (*p* < 0.001); this was true in early and late pregnancy. On comparing non-iodized salt consumption rate between the two groups, women with positive thyroid antibodies were more likely to consume non-iodized salt, and to have a significantly higher prevalence of previous thyroid disease, with the same results obtained in early (*p* = 0.002) and middle (*p* < 0.001) pregnancy. All antibody-positive pregnant women consumed less iodine-rich foods (*p* < 0.001) and iodine-rich nutritional supplement (*p* < 0.001), and thus, their iodine intake was lower (*p* < 0.001). Table 3 shows the general characteristics in terms of TPOAb/TgAb/TRAb positivity after removing the subjects with a previous thyroid history. Relative to unremoved historical data, pregnant women with thyroid autoimmunity had significantly lower UIC than antibody-negative individuals (*p* < 0.001).

**Table 2:** General Characteristics of Participants With and Without TPOAb/TgAb/TRAb(+), n(%)

	Pooled		Early pregnancy		Middle pregnancy		Later pregnancy	
	TPOAb/TgAb/TRAb(+)	TPOAb/TgAb/TRAb(−)	TPOAb/TgAb/TRAb(+)	TPOAb/TgAb/TRAb(−)	TPOAb/TgAb/TRAb(+)	TPOAb/TgAb/TRAb(−)	TPOAb/TgAb/TRAb(+)	TPOAb/TgAb/TRAb(−)
***N***	1439	3196	465	1072	617	1315	357	809
**Age, years**	29 (26–32)	29 (26–32)	29 (27–33)	29 (27–32)*	29 (26–32)	29 (26–32)	29 (26–32)	29 (26–33)
≥ 35	193 (13.41)	448 (14.02)	67 (14.41)	158 (14.74)	77 (12.48)	166 (12.62)	49 (13.73)	124 (15.33)
**Education status**								
≤ 9 years	481 (33.52)	977 (30.64)	112 (24.14)	257 (24.06)	242 (39.41)	435 (33.13)*	127 (35.57)	285 (35.27)
Senior high school and college	330 (23.00)	809 (25.37)	123 (26.51)	282 (26.40)	134 (21.82)	351 (26.73)*	73 (20.45)	176 (21.78)
Bachelor’s degree and higher	624 (43.48)	1403 (43.09)	229 (49.35)	529 (49.53)	238 (38.76)	527 (40.14)*	157 (43.98)	347 (42.95)
**Occupation status**								
Mental	1178 (81.86)	2525 (79.01)*	370 (79.57)	834 (77.80)	370 (79.57)	834 (77.80)	302 (84.59)	633 (78.24)*
Physical	261 (18.14)	671 (20.99)*	95 (20.43)	238 (22.20)	95 (20.43)	238 (22.20)	55 (15.41)	176 (21.76)*
**Family income per person last years, yuan**								
< 100,000	278 (19.41)	542 (17.02)*	69 (14.94)	145 (13.58)	131 (21.37)	240 (18.32)*	78 (21.85)	157 (19.48)
100,000–200,000	605 (42.25)	1296 (40.70)*	178 (38.53)	451 (42.23)	271 (44.21)	530 (40.46)*	156 (43.70)	315 (39.08)
≥ 200,000	549 (38.34)	1346 (42.27)*	215 (46.54)	472 (44.19)	211 (34.42)	540 (41.22)*	123 (34.45)	334 (41.44)
**Former smoker**	42 (2.93)	76 (2.38)	13 (2.81)	28 (2.62)	20 (3.26)	33 (2.51)	9 (2.52)	15 (1.85)
**Former drinker**	157 (11.07)	323 (10.13)	45 (9.80)	109 (10.21)	73 (11.99)	144 (10.97)	39 (11.14)	70 (8.65)
**First pregnancy,**	744 (51.70)	1640 (51.31)	225 (48.39)	519 (48.41)	336 (54.46)	704 (53.54)	183 (51.26)	417 (51.55)
**Previous thyroid disease**	214 (14.87)	293 (9.17)*	85 (18.28)	114 (10.63)*	86 (13.94)	118 (8.97)*	43 (12.04)	61 (7.54)
**Consumed iodine-rich food in the past 3 months**	1060 (73.66)	2610 (81.66)*	329 (70.75)	827 (77.15)*	460 (74.55)	1103 (83.88)*	271 (75.91)	680 (84.05)*
**Consumed iodized nutritional supplements in the past 3 months**	133 (9.24)	585 (18.30)*	53 (11.40)	156 (14.56)	49 (7.94)	251 (19.09)*	31 (8.68)	178 (22.00)*
**Non-iodized salt**	428 (29.74)	771 (24.12)*	145 (31.18)	274 (25.56)*	184 (29.82)	309 (23.50)*	99 (27.73)	188 (23.24)
**UIC, μg/L**	125 (73.85–200.69)	143.18 (84.92–224.95)*	134.18 (75.57–216.03)	153.86 (91.14–248.46)*	129.97 (79.71–204.88)	141.18 (85.64–221.00)	110.69 (63.84–185.36)	130.00 (71.60–201.39)*
**Iodine from iodine-rich food and salt, median(q1-q3), μg**	95.41 (23.81–180.00)	123.36 (42.36–220.42)*	73.02 (9.07–157.6)	103.22 (32.68–200.27)*	107.24 (39.58–182.87)	131.19 (46.90–226.43)*	109.34 (29.33–188.14)	137.07 (48.05–238.13)*
**Iodine-intake groups, μg**								
< 37.15	422 (29.32)	737 (23.06)*	176 (37.85)	279 (26.03)*	148 (23.99)	286 (21.75)*	98 (27.45)	172 (21.26)*
37.15 ~ 113.54	380 (26.41)	778 (24.34)*	125 (26.88)	292 (27.24)*	167 (27.071%)	306 (23.27)*	88 (24.65)	180 (22.25)*
113.54 ~ 205.99	370 (25.71)	789 (24.69)*	92 (19.78)	243 (22.67)*	185 (29.98)	347 (26.39)*	93 (26.05)	199 (24.60)*
≥ 205.99	267 (18.55)	892 (27.91)*	72 (15.8)	258 (24.07)*	117 (18.96)	376 (28.596%)*	78 (21.85)	258 (31.89)*
**Hypothyroidism**	42 (2.92)	79 (2.47)	15 (3.23)	25 (2.33)	15 (2.43)	32 (2.43)	12 (3.36)	22 (2.72)

Data are summarized as the median values (inter-quartile range) for continuous variables, or as numbers with proportions for categorical variables. Urine iodine concentration, UIC

*p < 0.05 versus the TPOAb/TgAb/TRAb(−) group

**Table 3:** General Characteristics of Participants With and Without TPOAb/TgAb/TRAb(+) - Without previous thyroid disease, n(%)

	Pooled		Early pregnancy		Middle pregnancy		Late pregnancy	
	TPOAb/TgAb/TRAb(+)	TPOAb/TgAb/TRAb(−)	TPOAb/TgAb/TRAb(+)	TPOAb/TgAb/TRAb(−)	TPOAb/TgAb/TRAb(+)	TPOAb/TgAb/TRAb(−)	TPOAb/TgAb/TRAb(+)	TPOAb/TgAb/TRAb(−)
***N***	1225	2903	380	958	531	1197	314	748
**Age, years**								
≥ 35*	154 (12.57)	393 (13.54)	49 (12.89)	139 (14.51)	62 (11.68)	143 (11.95)	43 (13.69)	111 (14.84)
**Education status**								
≤ 9 years	447 (36.61)	941 (32.48)*	106 (27.97)	243 (25.47)	222 (42.05)	419 (35.03)*	119 (37.90)	279 (37.35)
Senior high school and college	293 (24.00)	753 (25.99)*	106 (27.97)	263 (27.57)	120 (22.73)	327 (27.34)*	67 (21.34)	163 (21.82)
Bachelor’s degree and higher	481 (39.39)	1203 (41.53)*	167 (44.06)	448 (46.96)	186 (35.23)	450 (37.63)*	128 (40.76)	305 (40.83)
**Occupation status**								
Mental	1003 (81.88)	2286 (78.75)*	303 (79.74)	743 (77.56)	434 (81.73)	959 (80.12)	266 (84.71)	584 (78.07)
Physical	222 (18.12)	617 (21.75)*	77 (20.26)	215 (22.44)	97 (18.27)	238 (19.88)	48 (15.29)	164 (21.93)
**Family income per person last years, yuan**								
< 100,000	274((20.28)	514 (17.77)*	58 (15.38)	136 (14.26)	117 (22.20)	226 (18.94)*	72 (22.93)	152 (20.38)
100,000–200,000	525 (43.10)	1195 (41.31)*	153 (40.58)	405 (42.45)	233 (44.21)	492 (41.24)*	139 (44.27)	298 (39.95)
≥ 200,000	446 (36.52)	1184 (40.93)*	166 (44.03)	413 (43.29)	177 (33.59)	475 (39.82)*	103 (32.08)	296 (39.68)
**Former smoker**	35 (2.87)	70 (2.42)	10 (2.65)	25 (2.62)	17 (3.23)	31 (2.59)	8 (2.55)	14 (1.87)
**Former drinker**	133 (11.02)	293 (10.11)	34 (9.04)	96 (10.06)	63 (12.05)	129 (10.79)	36 (11.69)	68 (9.09)
**First pregnancy**	1488 (51.26)	634 (51.76)	466 (48.64)	186 (48.95)	288 (54.24)	636 (53.13)	160 (50.96)	386 (51.60)
**Consumed iodine-rich food in the past 3 months**	930 (75.92)	2383 (82.09)*	284 (74.74)	743 (77.56)	406 (76.46)	1011 (84.46)	240 (76.43)	629 (84.09)*
**Consumed iodized nutritional supplements in the past 3 months**	82 (6.69)	516 (17.77)*	27 (7.11)	134 (13.99)*	32 (6.03)	222 (18.55)*	23 (7.32)	160 (21.93)
**Non-iodized salt**	331 (27.02)	651 (22.43)*	102 (26.84)	222 (23.17)	146 (27.50)	263 (21.97)*	83 (26.43)	166 (22.19)
**UIC, μg/L**	124.57 (73.79–199.77)	143.67 (85.93–225.52)*	135.87 (73.39–219.33)	154.03 (91.40–246.95)*	128.93 (79.71–204.42)	142.23 (88.09–224.27)*	110.88 (64.30–186.60)	130.84 (71.29–201.67)*
**Iodine from iodine-rich food and salt, median(q1-q3),** μg	94.51 (25.26–180.00)	124.56 (44.98–221.53)*	69.48 (9.02–146.85)	104.86 (34.33–200.61)*	107.25 (42.55–185.07)	133.98 (51.56–228.61)*	109.44 (230.53–188.57)	137.60 (47.72–241.05)*
**Iodine-intake groups, μg**								
< 37.15	358 (29.22)	644 (22.18)*	148 (38.95)	243 (25.37)*	126 (23.73)	243 (20.30)*	84 (26.75)	158 (21.12)*
37.15 ~ 113.54	334 (27.27)	713 (24.56)*	108 (28.42)	260 (27.14)*	146 (27.50)	288 (24.06)*	80 (25.48)	165 (22.06)*
113.54 ~ 205.99	300 (24.49)	726 (25.01)*	68 (17.89)	224 (23.38)*	154 (29.00)	318 (26.57)*	78 (24.84)	184 (24.60)*
≥ 205.99	233 (19.02)	820 (28.25)*	56 (14.74)	231 (24.11)*	105 (19.07)	348 (29.07)*	72 (22.93)	241 (32.22)*
**Hypothyroidism, %**	30 (2.45)	63 (2.17)	13 (3.42)	20 (2.09)	10 (1.88)	24 (2.01)	7 (2.23)	19 (2.54)

Data are summarized as the median values (inter-quartile range) for continuous variables, or as numbers with proportions for categorical variables. Urine iodine concentration, UIC

*p < 0.05 versus the TPOAb/TgAb/TRAb(−) group

### Associations of type of salt, iodine-rich food consumption, iodized nutritional supplements with thyroid autoimmunity stratified by gestational week

**Fig. 3:**
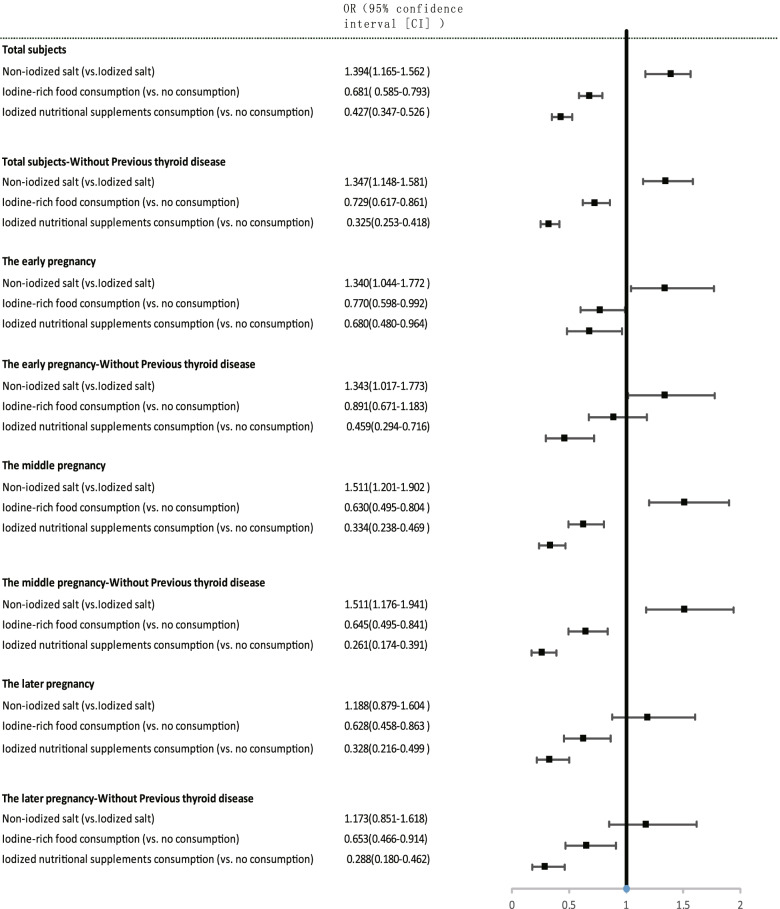
Adjusted associations of type of salt, iodine-rich food consumption, and iodized nutritional supplements with autoimmune thyroid disease stratified by gestational week. Data are reported as odds ratios (confidence intervals). Binary logistic regression analyses (forward stepwise) were performed. Adjustments for age, educational status, current smoker, current drinker, first pregnancy, and previous thyroid disease were also performed

**Fig. 4:**
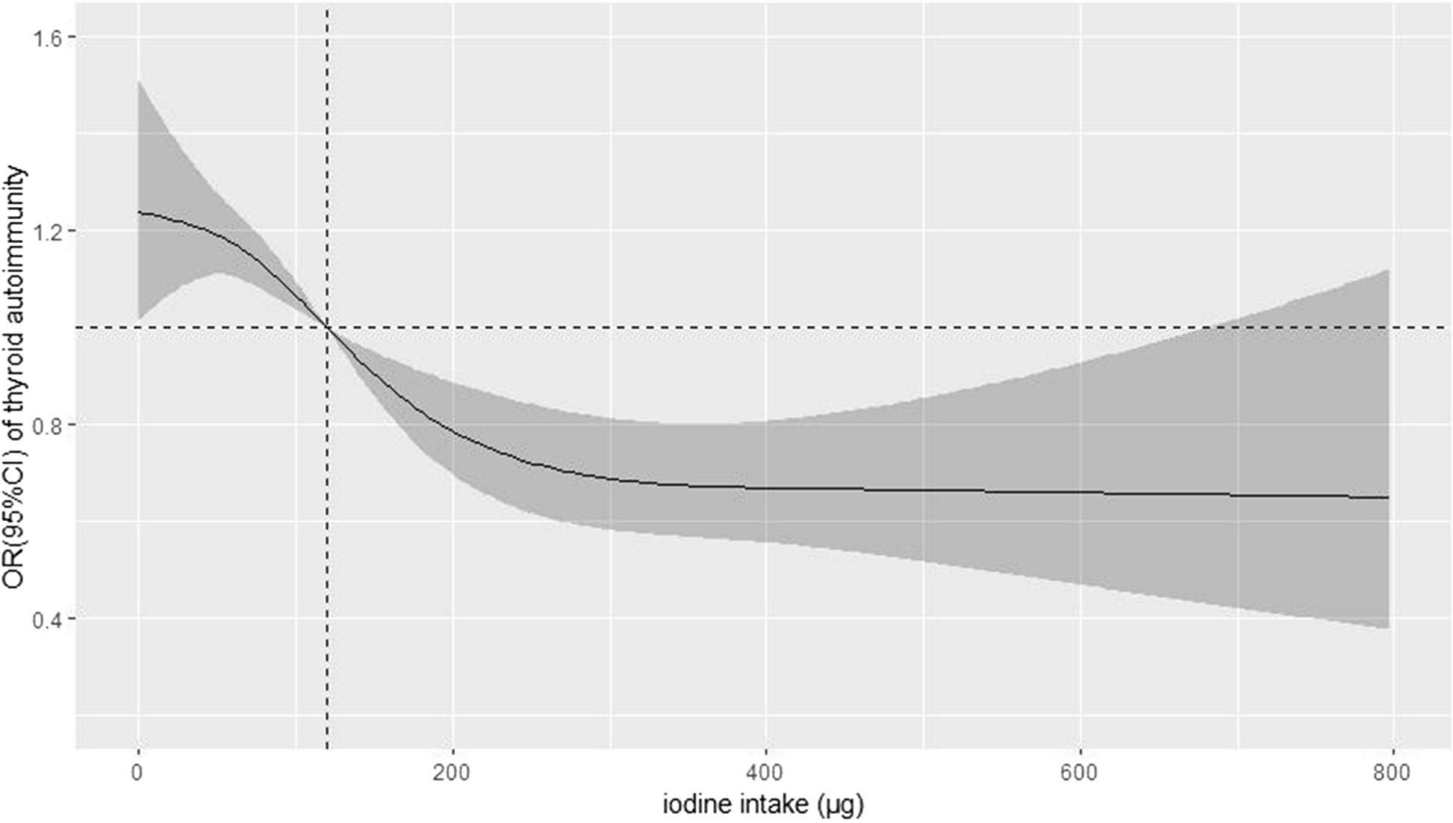
Association between iodine intake and the risk of thyroid autoimmunity, allowing for nonlinear effects, with 95% CI. Data are reported as odds ratios (confidence intervals). Binary logistic regression analyses were performed. Adjustments for age, educational status, current smoker, current drinker, first pregnancy, and previous thyroid disease were also performed

The associations of non-iodized salt, iodine-rich food consumption, and iodized nutritional supplements with thyroid autoimmunity were estimated (Figs. 3 and 4). After adjusting for age, educational status, former smoker, former drinker, first pregnancy, and previous thyroid disease, non-iodized salt (OR = 1.394 [CI 1.165–1.562]; *p* = 0.003), iodine-rich food consumption (OR = 0.681 [CI 0.585–0.793]; *p* = 0.003), and iodized nutritional supplements (OR = 0.427 [CI 0.347–0.526]; *p* = 0.003), were individually associated with thyroid autoimmunity in all participants. However, in the gestational week-specific analysis, no association was found between non-iodized salt and thyroid autoimmunity in late pregnancy, and between iodine-rich food consumption and thyroid autoimmunity in early pregnancy after removing subjects with previous thyroid disease. Other associations were still statistically significant.

### Dose-response relationship between iodine intake (iodine from iodine-rich food, iodized salt, and iodized nutritional supplements) and thyroid autoimmunity

The overall median iodine intake (25th and 75th percentiles) was 103.6 (31.1–197.0) μg/day, of which only 20.5% greater or equal to 230 μg/day indicating adequate iodine intake. We used an RCS model with 4 knots to simulate the relationship between iodine intake and the risk for thyroid autoimmunity. A significant nonlinear dose-response association was found in the relationship between iodine intake and the risk for thyroid autoimmunity (*p* = 0.019). This analysis showed that with the continuous change in iodine intake, the association strength of thyroid autoimmunity decreased nonlinearly (Fig. 4).

### Associations of UIC with thyroid autoimmunity

The association between UIC and thyroid autoimmunity is shown in Table 4. After multivariable adjustment, in comparison with participants with adequate iodine nutrition, those with iodine deficiency (lower UIC) had an increased risk of TPOAb/TgAb/TRAb (+) (OR = 1.324 [CI 1.125–1.559]; *p* < 0.001). However, when the subjects were stratified by gestational week, those associations were observed only in early (OR = 1.364 [CI 1.023–1.819]; *p* = 0.039) and late (OR = 1.701 [CI 1.213–2.384]; *p* = 0.021) pregnancy. Meanwhile, in late pregnancy, subjects with low (OR = 1.512 [CI 1.027–2.226]; *p* = 0.036) and excessive (OR = 2.455 [CI 1.001–5.377]; *p* = 0.049) UIC also had a higher risk of TPOAb/TgAb/TRAb (+) status. Moreover, even after removing subjects with a previous thyroid disease analysis, these risk factors remained.

**Table 4:** Associations of Urinary Iodine Concentration with thyroid autoimmunity (TPOAb/TgAb/TRAb(+)) Stratified by Gestational Week

Urinary iodine	TPOAb/TgAb/TRAb(+)	OR [CI]	***p***-value	TPOAb/TgAb/TRAb(+) (without previous thyroid disease)	OR [CI]	***P***-value
**Pooled**						
Lower (UIC ≤ 100 μg/L)	34.73	**1.324 (1.125–1.559)**	**0.0007**	33.81	**1.342 (1.127–1.599)**	**0.0010**
Low (100 μg/L < UIC < 150 μg/L)	32.36	1.184 (0.983–1.425)	0.0747	30.64	1.151 (0.943–1.405)	0.1662
Adequate (150 μg/L ≤ UIC < 250 μg/L)	28.76	1.00		27.64	1.00	
More than adequate (250 μg/L ≤ UIC < 500 μg/L)	26.76	0.906 (0.735–1.117)	0.3567	24.53	0.857 (0.684–1.075)	0.1817
Excessive (UIC ≥ 500 μg/L)	23.46	0.746 (0.506–1.101)	0.1402	22.30	0.740 (0.489–1.120)	0.1544
**Early pregnancy**						
Lower	35.11	**1.364 (1.023–1.819)**	**0.0392**	33.88	**1.370 (1.005–1.867)**	**0.0468**
Low	31.51	1.164 (0.836–1.620)	0.4537	27.24	0.998 (0.691–1.442)	0.9912
Adequate	28.61	1.00		27.40	1.00	
More than adequate	25.96	0.885 (0.626–1.250)	0.4911	24.80	0.895 (0.616–1.301)	0.5607
Excessive	17.19	0.479 (0.235–0.974)	0.0402	14.29	0.391 (0.171–0.893)	0.0260
**Middle pregnancy**						
Lower	34.49	1.147 (0.894–1.472)	0.2812	33.87	1.153 (0.844–1.503)	0.2938
Low	32.55	1.045 (0.791–1.379)	0.7581	31.32	1.015 (0.755–1.365)	0.9220
Adequate	31.38	1.00		30.87	1.00	
More than adequate	27.78	0.867 (0.627–1.199)	0.3883	24.90	0.727 (0.510–1.035)	0.0767
Excessive	21.92	0.615 (0.342–1.105)	0.1038	21.74	0.629 (0.343–1.155)	0.1347
**Later pregnancy**						
Lower	34.65	**1.701 (1.213–2.386)**	**0.0021**	33.66	**1.811 (1.260–2.640)**	**0.0013**
Low	33.06	1.512 (1.027–2.226)	0.0363	33.19	1.711 (1.137–2.573)	0.0099
Adequate	24.13	1.00		22.14	1.00	
More than adequate	24.68	1.042 (0.661–1.670)	0.8361	23.40	1.122 (0.686–1.837)	0.6460
Excessive	44.00	**2.455 (1.001–5.377)**	**0.0499**	43.48	**2.584 (1.071–6.235)**	**0.0346**

Data are odds ratios [confidence intervals]. Binary logistic regression analyses (forward stepwise) were performed. Adjustment for age, educational status, occupation status, family income in the last year, current smoker, current drinker. Urinary iodine concentrations: lower, < 100 μg/L; low, 100 to < 150 μg/L; adequate, 150 to < 250 μg/L; more than adequate, 250 to < 500 μg/L; excessive, ≥500 μg/L. *OR* Odds ratio, *CI* Confidence interval

## Discussion

In this study, we analyzed a large sample size of pregnant women to study the relationship between iodine nutrition during pregnancy and thyroid autoimmunity in Shanghai, China. We found that 25.9% of the participants consumed non-iodized salt, and 54.5% had ID, and 31.0% had thyroid autoimmunity. The consumption of non-iodized salt and the presence of lower UIC were significantly associated with a higher prevalence of TPOAb/TgAb/TRAb (+). Sufficient iodine intake and good iodine nutrition, such as consumption of iodized salt, iodine-rich foods, and iodine nutritional supplements, were negatively correlated with TPOAb/TgAb/TRAb (+); with the continuous changes in iodine intake, the strength of the association with thyroid autoimmunity decreased nonlinearly. These findings indicate that insufficient iodine intake may contribute to the high proportion of ID and may be associated with an increased risk of thyroid autoimmunity.

Iodine requirements are greater during pregnancy, predominantly because of increased renal iodide clearance and the use of iodine for thyroid hormone production. In 2017, the American Thyroid Association guidelines suggested that “all pregnant women should ingest approximately 250 μg iodine daily” and recommended “all pregnant and lactating women should supplement their diet with a daily oral iodine supplement of 150 μg/d irrespective of historical iodine nutrition status” [25, 26]. Chinese dietary reference intake (2013) indicated that the recommended intake of iodine for pregnant women was 230 μg/d [24]. After implementation of the USI program for two decades, the Chinese achieved the goal of elimination of IDDs, but recent studies showed that the population in pregnancy was generally iodine-deficient in many provinces [27–29]. Wang et al. reported that pregnant women in Shanghai also had mild iodine deficiency [13, 14]. In fact, pregnant women show widespread iodine deficiency in developing and developed countries around the world [30–34]. Our study found that pregnant women who consumed iodized salt had better iodine nutrition than who consumed non-iodine salt (UIC: 141.0 [83.4–221.4] and 129.5 [74.0–209.1], *p* < 0.001). A study conducted in Zhejiang Province of China also demonstrated similar findings where consuming adequately iodized salt seemed to increase the median UIC level (159 [97.6–215.0] and 99.6 [62.2–168.0], *p* < 0.001) [35].

Our study shows that consumption of iodized salt, iodine-rich food, and iodized nutritional supplements was negatively correlated with thyroid autoimmunity in pregnant women, and nonlinear dose-response association analysis showed that with continuous changes in iodine intake, the association strength with thyroid autoimmunity decreased nonlinearly. When excluding those with previous thyroid disease, this protection effects were still valid. The study by Chen et al. also found that consumption of iodized salt might be a protective factor for thyroid autoimmunity, especially for women^36.^ However, Rayman et al. reported that chronic exposure to excess iodine intake induces thyroid autoimmunity, and appropriate status of iodine is crucial to thyroid health [36]. Both iodine deficiency and excess have been reported to be associated with increased positivity rates for thyroid autoantibodies. Iodine deficiency may influence thyroid autoimmunity through inflammatory response induced by oxidative stress [37]. In contrast, iodine excess occurs partly because highly-iodinated thyroglobulin (Tg) is more immunogenic [38].

Approximately 90% of ingested iodine is excreted through the kidneys, so UIC is an excellent biochemical marker for the evaluation of recent iodine intake [38]. In the present study, our findings showed that lower UIC (≤100 μg/L) was an independent risk factor of thyroid autoimmunity in pregnant women, those associations were observed in early and late pregnancy. The same conclusion came from Sun Jie who conducted a cross-sectional study of early pregnant women [39]. In contrast, Shi Xiaoguang reported that the prevalence of TPOAb positivity and TgAb positivity presented a U-shaped curve, ranging from mild iodine deficiency to iodine excess in a cross-sectional study of 7190 pregnant women in China [40]. Our study found the similar result only in late pregnant women. In the current studies, there are few reports about the relation between the iodine and thyroid antibodies in middle and late pregnant women. Further research is needed to clarify the significance of these effects in middle and late of pregnancy.

The strengths of our study are as follows: this cross-sectional study was the largest scale survey on the relationship between the prevalence of thyroid autoimmunity and iodine intake during the entire pregnancy. When the whole nutritional status of the population changes dramatically in Shanghai so far, the biological function of iodine changed accordingly [13, 14]. In these circumstances, we analyzed the correlation between thyroid autoimmunity and iodized salt and UIC with/without previous thyroid disease, and obtained consistent conclusions, which indicate that our study results were reliable and stable. The limitation of our study was that cross-sectional epidemic research could not establish a causal relationship. Our conclusion is drawn from the current nutritional status of pregnant women in Shanghai, and extrapolation needs to be performed with caution.

## Conclusion

In summary, there is a high prevalence of ID in pregnant women in Shanghai, and inadequate iodine nutrition of pregnant women is a risk factor for thyroid autoimmunity. Nutritional status of iodine in pregnant women is not well recognised in Shanghai. The use of iodised salt and supplementation of iodine-rich nutrients during pregnancy are recommended.

## Data Availability

Please contact author for data requests.

## Abbreviations

TPOAb: Thyroid peroxidase antibodies
TgAb: Hyroglobulin antibodies
TRAb: Thyrotrophic antibodies
IQR: Interquartile range
CI: Confidence interval
UIC: Urinary iodine concentration
IDDs: Iodine deficiency disorders
ID: Iodine deficiency
USI: Universal salt iodization
SCDC: Shanghai Municipal Center for Disease Control and Prevention
FFQ: Food frequency questionnaire
TSH: Serum thyrotropin
TPO: Thyroid peroxidase
Tg: Thyroglobulin
TR: Thyrotrophic receptor
RCS: Restricted cubic spline
